# Evaluation of TgH(CX3CR1-EGFP) mice implanted with mCherry-GL261 cells as an in vivo model for morphometrical analysis of glioma-microglia interaction

**DOI:** 10.1186/s12885-016-2118-3

**Published:** 2016-02-08

**Authors:** Fernando F. B. Resende, Xianshu Bai, Elaine Aparecida Del Bel, Frank Kirchhoff, Anja Scheller, Ricardo Titze-de-Almeida

**Affiliations:** Laboratório de Tecnologias para Terapia Gênica, ASS 128, ICC Sul, Universidade de Brasília-UnB, Campus Darcy Ribeiro, FAV., Brasília, DF Brasil 70910-970; Molecular Physiology, Center for Integrative Physiology and Molecular Medicine (CIPMM), University of Saarland, Homburg, Germany; Laboratório de Neurofisiologia e Biologia Molecular, Department Morfologia Fisiologia e Patologia Básica, FORP, Universidade de São Paulo - USP, Ribeirão Preto, SP Brasil 14040-904

**Keywords:** Microglia, Glioma, GL261, CX3CR1, Cancer, Morphology, 2P-LSM

## Abstract

**Background:**

Glioblastoma multiforme is the most aggressive brain tumor. Microglia are prominent cells within glioma tissue and play important roles in tumor biology. This work presents an animal model designed for the study of microglial cell morphology in situ during gliomagenesis. It also allows a quantitative morphometrical analysis of microglial cells during their activation by glioma cells.

**Methods:**

The animal model associates the following cell types: 1- mCherry red fluorescent GL261 glioma cells and; 2- EGFP fluorescent microglia, present in the TgH(CX3CR1-EGFP) mouse line. First, mCherry-GL261 glioma cells were implanted in the brain cortex of TgH(CX3CR1-EGFP) mice. Epifluorescence − and confocal laser-scanning microscopy were employed for analysis of fixed tissue sections, whereas two-photon laser-scanning microscopy (2P-LSM) was used to track tumor cells and microglia in the brain of living animals.

**Results:**

Implanted mCherry-GL261 cells successfully developed brain tumors. They mimic the aggressive behavior found in human disease, with a rapid increase in size and the presence of secondary tumors apart from the injection site. As tumor grows, mCherry-GL261 cells progressively lost their original shape, adopting a heterogeneous and diffuse morphology at 14–18 d. Soma size increased from 10–52 μm. At this point, we focused on the kinetics of microglial access to glioma tissues. 2P-LSM revealed an intense microgliosis in brain areas already shortly after tumor implantation, i.e. at 30 min. By confocal microscopy, we found clusters of microglial cells around the tumor mass in the first 3 days. Then cells infiltrated the tumor area, where they remained during all the time points studied, from 6–18 days. Microglia in contact with glioma cells also present changes in cell morphology, from a ramified to an amoeboid shape. Cell bodies enlarged from 366 ± 0.0 μm^2^, in quiescent microglia, to 1310 ± 146.0 μm^2^, and the cell processes became shortened.

**Conclusions:**

The GL261/CX3CR1 mouse model reported here is a valuable tool for imaging of microglial cells during glioma growth, either in fixed tissue sections or living animals. Remarkable advantages are the use of immunocompetent animals and the simplified imaging method without the need of immunohistochemical procedures.

## Background

Previous studies about glioma biology revealed that microglia is the dominant immune cell within tumor mass, accounting for about 30% of the tumor cell content [1]. Glioma and microglia exert reciprocal and pro-tumorigenic influences [2]. Glioma cells stimulate microglia to express genes that favor tumor growth. A well-known example is the membrane type-1 matrix metalloproteinase (MT1-MMP), that disrupts the extracellular matrix of brain tissues, opening anatomic spaces for tumor expansion [3]. Glioma cells also influence microglia to present an activated alternative phenotype; activated microglia in turn expresses pro-tumorigenic receptors and cytokines [4, 5].

Studies on the role of microglia in glioma development have taken advantage of animal models and innovative microscopy techniques [6–11]. The TgH(CX3CR1-EGFP) is a mouse strain in which the fractalkine receptor gene (CX3CR1) was replaced by the green fluorescent protein (GFP) reporter gene [12]. CX3CR1 is a seven-transmembrane G-protein-coupled receptor that plays a role in leukocyte migration and adhesion [13]. Peripheral blood monocytes, subsets of natural killer cells (NK) and dendritic cells, and also microglia naturally express CX3CR1 [12]. This transgenic mouse strain (C57BL/6 N background) can develop brain tumors when implanted with the GL261 cell line, an established model of glioblastoma multiforme [14]. GL261 cells do not require a suppressed immune system to generate tumors. Therefore, this feature simplifies the methodology and provides a model that represents various aspects of the human disease [15].

Advanced imaging techniques are also important to follow brain tumor development and microgliosis. Regarding brain imaging, two-photon laser-scanning microscopy (2P-LSM) has brought significant improvements in the fields of neuroscience and oncology. First, the method allows fluorescence imaging of cells and tissues from living animals [16]. Indeed, the three-dimensional images captured by 2P-LSM present a 100-fold increase in penetration depth compared with confocal microscopy. This advantage is especially important for images of brain tumors recorded in vivo [17, 18].

This study reports on a glioma model dedicated to the study of microglial cells in tumor tissues. The model comprises a fluorescent glioma cell, mCherry-GL261, that was implanted in the cortex of TgH(CX3CR1-EGFP) mice with fluorescent microglia. This GL261/CX3CR1 model allowed analysis of microglia morphology during tumor growth and morphometrical measure of parameters of microglia activation in tumor areas.

## Methods

### Ethics statement

This work was conducted at the University of Saarland in strict accordance to the European and German guidelines for the welfare of experimental animals under the license 65/2013, approved by the Saarland state’s “Landesamt fuer Gesundheit und Verbraucherschutz” in Saarbrücken/Germany.

### Cell culture

This study used the mCherry-GL261 tumor cells [19], a glioma cell line with expression of the fluorescent protein mCherry, kindly provided by Helmut Kettenmann (Max-Delbrück-Center for Molecular Medicine, Berlin, Germany). Cells were maintained on 75 cm^3^ culture flasks with DMEM/F12 [supplemented with 10 % (volume/volume) heat-inactivated fetal calf serum, and 1 % of penicillin/streptomycin solution, all obtained from Invitrogen (Karlsruhe, Germany)]. When the confluence reached about 90 %, the cells were harvested using 0.25 % Trypsin solution (Invitrogen, Karlsruhe, Germany). A pellet was formed by centrifugation at 1200 g for 3 min (Hettich Universal 30 F, Tuttlingen, Germany). Cells were diluted in 1.0 ml PBS and counted in Moxi-Z (Orflo, Hailey, USA). Aliquots containing 5x10^4^ cells in PBS were made and centrifuged at 1200 g for 3 min (Eppendorf MiniSpin, Hamburg, Germany). The pellet was re-suspended in 3 μL of PBS by gently mixing, and the 5 μL resulting solution was immediately injected into the mouse brain by using a microliter syringe (Hamilton) with a 25 gauge needle.

### Mouse line

Adult TgH(CX3CR1-EGFP) heterozygous mice [12] backcrossed to C57BL/6 N background for more than 10 generations were maintained at a temperature and light controlled animal facility, and received food and water *ad libitum*.

### Intracortical injections of mCherry-GL261 cells

All animals were anesthetized with Ketamine/Xylazine (Bayer, Germany) (140 mg/10 mg/kg body weight) by intraperitoneal injection. They had the top of head shaved, and the surgery site cleaned with iodine antiseptic. Bepanthen® cream (Bayer, Germany) was used to cover and protect the eyes. After proper mouse fixation in the stereotaxic instrument, a single skin cut of about 0.5 cm was performed by using scissors, followed by gently divulsion of the sub-cutaneous tissue. A 2.0 mm hand drill (Fine Science Tools, Heidelberg, Germany) was used to thin the skull at the injection site, approximately at 2.0 mm posterior and 1.5 mm lateral of the bregma. Five microliters of the mCherry-GL261 cell suspension (a total of 50.000 cells) were aspired with a micro-syringe and slowly injected, first 2.0 mm below skull surface into the cortex, and then the needle was pushed back 0.5 mm to inject the rest of the volume. About 5 min were spent to complete the injection and two additional min for totally removing of the needle. The sub-cutaneous tissue was gently washed with NaCl, 0.9 % and the skin closed with simple interrupted sutures. Iodine antiseptic was applied on the suture after the surgery to prevent infections. Immediately afterwards, the mice were kept on a heating plate until woken up. To release the pain, buprenorphine (0.09 g/30 g body weight) was administrated after the surgery and at the following day. Once the tumor cells were injected, the mice were housed individually in the same conditions as described before. For in vivo imaging, the procedure to inject the glioma cells was the same. However, after injection a cranial window was formed in the same area, to acquire the images as described below. Mice, which received injections of mCherry-GL261 cells, were daily monitored for weight and clinical status. If an animal’s weight dropped 15% below the baseline or became symptomatic, it was euthanized.

### Cranial window surgery

The procedures were carried out under anesthesia initiated by Ketamine/Xylazine (Bayer, Germany) (140 mg/10 mg/kg body weight). Afterwards mice were placed on a heating pad, heads were stabilized in a stereotactic frame using ear bars, artificial ventilation was continued with a gas mixture of O_2_ (50%), and N_2_O (50%) at 120 strokes/min (100–160 μl/stroke depending on the body weight). A longitudinal incision of the skin was performed between the occiput and the forehead. The subcutaneous membrane was completely removed. A 0.5 mm hand drill (Fine Science Tools, Heidelberg, Germany) was used to thin the skull and to open a 5.0 mm diameter window, approximately at 2.0 mm posterior and 1.5 mm lateral of the bregma. The dura mater was continuously rinsed with artificial cerebro-spinal fluid: 125 mM NaCl, 25 mM NaHCO_3_, 2.5 mM KCl, 1.25 KH_2_PO_4_, 1 mM MgCl_2_, 2 mM CaCl_2_*H_2_O and 10 mM glucose. To cover the window and make the surface flat, a 5.0 mm cover glass (Thermo-Scientific, Germany) was fixed by Self-Adhesive Resin Cement ESPE RelyX U200 (3 M, Seefeld, Germany). In addition, the head was rigidly fixed with a custom-made clamp. The rectal body temperature was measured and kept between 36 and 38 °C by a heating plate. The depth of anesthesia was tested by provoking the corneal reflex and reactions to *noxious stimuli*.

### Microscopic analysis

Photographic overview images were captured using an epifluorescence microscope Axio Imager Z2 (Zeiss, Oberkochen, Germany) equipped with a 5x objective. Confocal images were taken using a laser-scanning microscope LSM-710 (Zeiss, Oberkochen, Germany) with appropriate excitation and emission filters. Z-stack images were taken at 0.8–2.0 μm intervals and processed with the ZEN software (Zeiss, Oberkochen, Germany). All data were collected from three randomly selected pictures, from three different slices of at least three mice per group.

### Morphometric analysis of microglia based on skeletonization, measurement of cell length, soma size and Iba1 expression

This study carried out a morphometrical analysis of microglial cells present in brain tumor tissues (region 2, tumor border, and region 3, tumor core) in comparison with the contralateral non-inoculated hemisphere (region 1). First, the number of microglial cells was counted in each region. Data included confocal stacks (*n* = 14) of 0.8–2.0 μm intervals of each individual cell, present in three randomized images of each region, in four animals. In addition, an immunohistochemical detection of Iba1 (ionized calcium binding adaptor molecule 1), a marker of microglia / macrophage, was carried out. For that, slices were first treated with a blocking solution (0.3 % Triton X-100 and 5.0 % horse serum in PBS) for 1 h at room temperature (RT). They were incubated with primary antibody to Iba1 (1:500, polyclonal, rabbit–Wako Chemicals, Neuss, Germany) at 4.0 °C overnight, then washed 3 times in PBS. The fluorescent secondary antibody (AlexaFluor® 633–labeled anti-mouse IgG, Invitrogen, Grand Island NY, USA), diluted 1:2000 in 2.0 % horse serum in PBS, was incubated for 2 h at RT. After that, sequences of 14 stacks were evaluated by using the ZEN Software to determine the mean intensity value.

For skeletonization, first, images from brain regions were acquired with a confocal microscope. Maximum intensity projections were treated to remove background noise. The images were converted to binary, presented to skeletonization, and analyzed by the ImageJ plugin AnalyzeSkeleton [20]. The parameter end points express the complexity of microglial cell structure. It corresponds to branch ends, determined by voxels with less than two neighbors [21–23]. To measure the soma size, sequences of 14 stacks were examined with the ZEN Software (Zeiss, Oberkochen, Germany). Each microglial soma was marked with Spline contour tool, which revealed the area in μm^2^. Finally, the total length of each microglia was quantified, with the maximum intensity projections, by using the ImageJ plugin ROI manager.

### Two-photon laser-scanning microscopy and image acquisition

High resolution in vivo imaging was performed using a custom-made 2P-LSM equipped with fs-pulsed titanium-sapphire laser (Chameleon Ultra II; Coherent, USA). For 2P-recordings, a Zeiss W Plan Apochromat 20× (NA 1.0) water immersion objective was used. Laser excitation was set at 895 ± 5 nm for EGFP and mCherry detection. Emitted light was split by a 520 nm longpass dichroic mirror (Semrock, Rochester, USA) and collected by photo-multiplier tubes (Hamamatsu, Japan) through two bandpass filters: a 494 ± 20.5 nm (FF01-494/41-25) and a 542 ± 25 nm (FF01-542/50-25 (Semrock). In parallel, uniformly spaced (1.5–2.4 μm) planes of 100*100 to 600*600 μm^2^ regions were recorded, digitized and processed to obtain z-stacks of images (256 × 256 to 1024 × 1024 pixels in size). Voxel sizes ranged from 0.2 × 0.2 × 1.5 to 1.17 × 1.17 × 2.4 μm for the xyz-axes. Recordings of, at most, 100 μm stack depth were obtained.

### Statistical analysis

All data were analyzed by using the Statistical Package for Social Sciences (IBM SPSS Statistics for Windows, Version 20. Armonk, NY) and expressed as mean ± standard error of the mean. We used the one-way analysis of variance (ANOVA) followed by Tukey’s test to test intergroup differences. Differences between pairs of experimental groups were analyzed by the Student - t test. The level of statistical significance adopted was *p* < 0.05.

## Results

In this study, we evaluated a mouse model designed to monitor the microglial infiltration into tumor tissues and, in addition, being able to track changes in the morphology of microglia under glioma influence. All TgH(CX3CR1-EGFP) immunocompetent mice (*n* = 24) developed brain tumors within 3 to 18 days after intracortical injections of mCherry-GL261 glioma cells. This GL261/CX3CR1 model revealed the pattern of microglial infiltration into glioma tumor mass, in terms of changes in cell shape and counting, and provided data for morphometrical analysis of activated microglia.

### Analysis of microglial cells infiltration during glioma growth

**Fig. 1:**
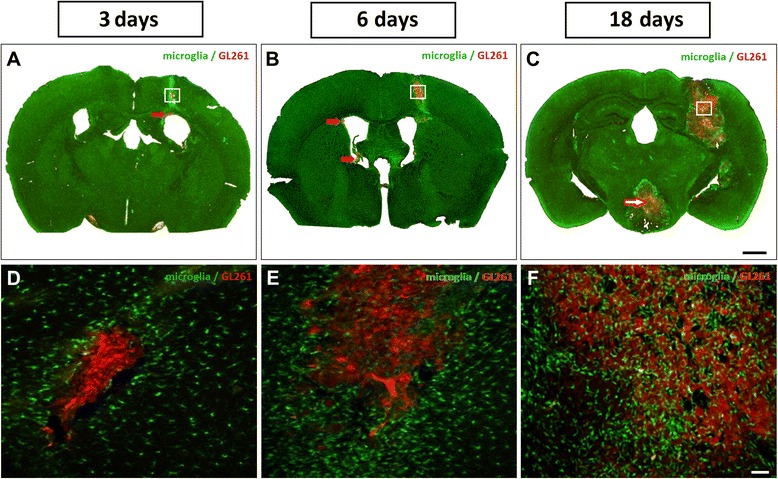
Epifluorescence imaging (**a**) 3, (**b**) 6 and (**c**) 18 dpi reveals tumor growth and metastasis. Coronal sections of a TgH(CX3CR1-EGFP) mouse brain implanted with mCherry-GL261 cells. **a** and **d** Glioma cells infiltrated the cortex and surrounding areas; red arrow (**a**) points to a secondary tumor in the left ventricle. Injection site three dpi of mCherry-GL261 cells, showing an intense microgliosis around the tumor tissue. **b** and **e** red arrows (**b**) point to secondary tumors in the right ventricle. Microglial infiltration in tumor core 6 days after injection, while microgliosis remained present in tumor border. **c** and **f** An enlarged and diffuse mass of glioma cells developed 18 dpi, including metastasis in the hypothalamus (*red outlined arrow*, **c**). Epifluorescence imaging in (**a**, **b** and **c**), confocal microscopy of the region indicated by white boxes in (**a**–**c**) in (**d**, **e** and **f**). Scale bars: (**a**–**c**), 500 μm; (**d**–**f**), 100 μm

First, our GL261/CX3CR1 model allowed imaging of microglia and glioma cells, easily discriminated by specific fluorescences. Intracortically implanted mCherry-GL261 cells formed clusters of red-fluorescent glioma cells, as shown in Fig. 1. Green-fluorescent microglia interacted with these tumors, as examined at 3, 6 and 18 days post injection (dpi). Microglial cells presented a time- and space-dependent pattern of infiltration into developing tumor areas. In sections examined three days after inoculation, we identified microglial cells next to mCherry-GL261 cells (Fig. 1a, d). The density of microglia close to developing glioma was higher in comparison with other brain regions. Indeed, all cells avoided to cross the tumor borders. However, six days after tumor injection, many activated microglial cells had infiltrated the tumor mass (Fig. 1b, e). Some activated microglia also remained growing around the tumor, in close contact with glioma cells. At 18 dpi, we found an increased number of microglial cells into the enlarged and diffuse mass of glioma cells (Fig. 1c, f).

The mCherry-GL261 cells used in our model mimicked the typical aggressive behavior also reported in patients with glioblastoma multiforme. Tumor cells rapidly grew and infiltrated surrounding tissues. As shown in Fig. 1c, at day 18 the tumor mass compressed the left hippocampus, deforming regions CA1, CA2, CA3, and the dentate gyrus. Implanted mCherry-GL261 cells also developed secondary tumors apart from the main tumor mass, formed by cell migration or metastasis. They were present in the border of left lateral ventricle in the early 3 dpi (Fig. 1a, red arrow). Secondary tumors also appeared at 6 dpi in the margins of the right lateral ventricle (Fig. 1b, red arrows). Finally, a more prominent mass apart from the injection site developed next to hypothalamus at 18 dpi (Fig. 1c, red outlined arrow).

### Tracking microglial morphological changes and quantitative cell analysis during their infiltration into the tumor mass

**Fig. 2:**
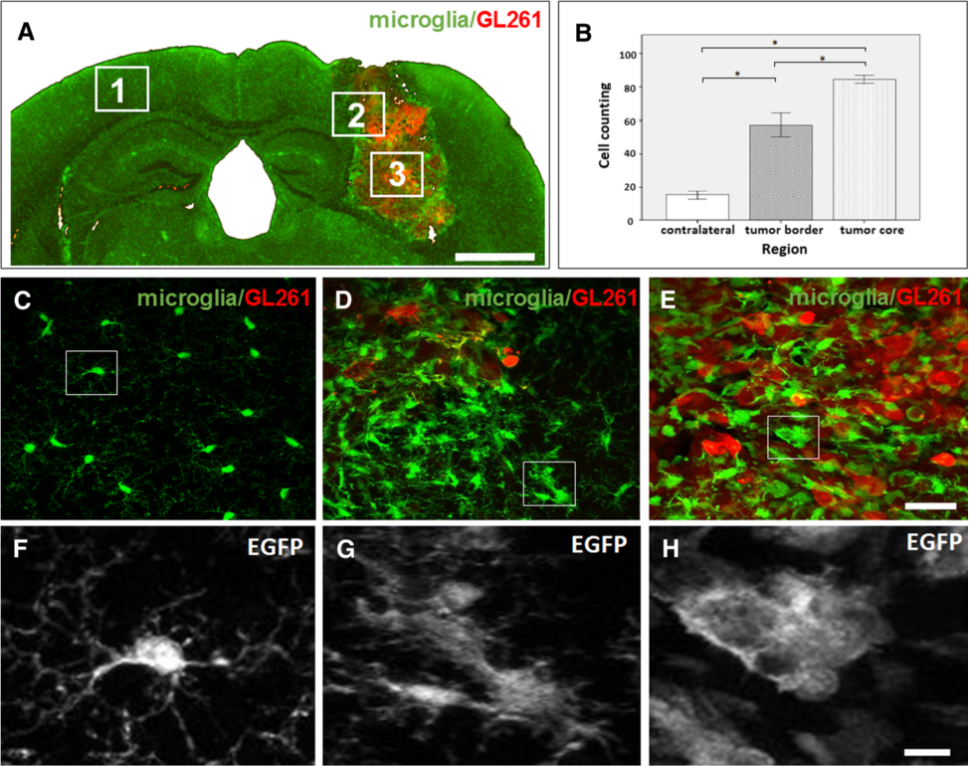
Analysis of microglial cells shows morphological changes depending on their location towards the tumor tissue. **a** Coronal section of a TgH(CX3CR1-EGFP) mouse brain implanted with mCherry-GL261 cells at 14 dpi. White boxes indicate the following regions in analysis: 1 − contralateral hemisphere (control non-implanted site); 2 − tumor border and; 3 − tumor core. **b** Quantitative analysis of microglia cell numbers in regions 1–3, showing an increased number of microglial cells in tumor regions 2 and 3 (border and core, respectively) in comparison with the control region 1 (*p* < 0,05). In addition the number increase also in the core compared to the border tumor region. **c**–**e** Higher magnification of the brain areas 1–3 (white squares) in (**a**); maximum intensity projections acquired by confocal microscopy. **f**–**h** Magnified views of single cells (white boxes in **c**–**e**) to show details in cell morphology. In the presence of glioma cells, microglial morphology changes from a ramified (**c** and **f**) to an amoeboid shape in the tumors border (**d** and **g**) and core (**e** and **h**). Scale bars: (**a**), 500 μm; (**c**–**e**), 20 μm; (**f**–**h**), 10 μm

The present GL261/CX3CR1 glioma model also allowed imaging of microglial cells present in tumor areas for analysis of morphology and cell density. Confocal microscopy successfully captured cell fluorescences, organized in stacks of images from fixed brain slices mounted on glass slides. We first focused on cells present in non-inoculated brain regions, like region 1 (Fig. 2a, c, and f). These surveilling microglia showed a ramified morphology, with a small cell body and fine processes. In contrast, cells near the tumor regions assumed an amoeboid shape, a sign of microglial activation. Cell bodies were enlarged and the cellular processes displayed reduced lengths and ramifications. Amoeboid cells were present either on the borders as shown in region 2 (Fig. 2a, d, and g) or infiltrated into the tumor core, region 3 (Fig. 2a, e, and h) at 14 dpi.

Our model also allowed to quantify microglial cell numbers in regions 1, 2, and 3. Both tumor regions (border and core) presented a higher number of microglial cells in comparison with the contralateral brain hemisphere (Fig. 2b, *p* < 0.05). In addition, cell density was significantly higher in the core region of tumors compared to the borders. These results reinforced the notion that glioma cells recruit microglia and induce its activation.

### Longitudinal measure of microglial activation and morphometrical analysis of microglia during the infiltration into tumor mass

**Fig. 3:**
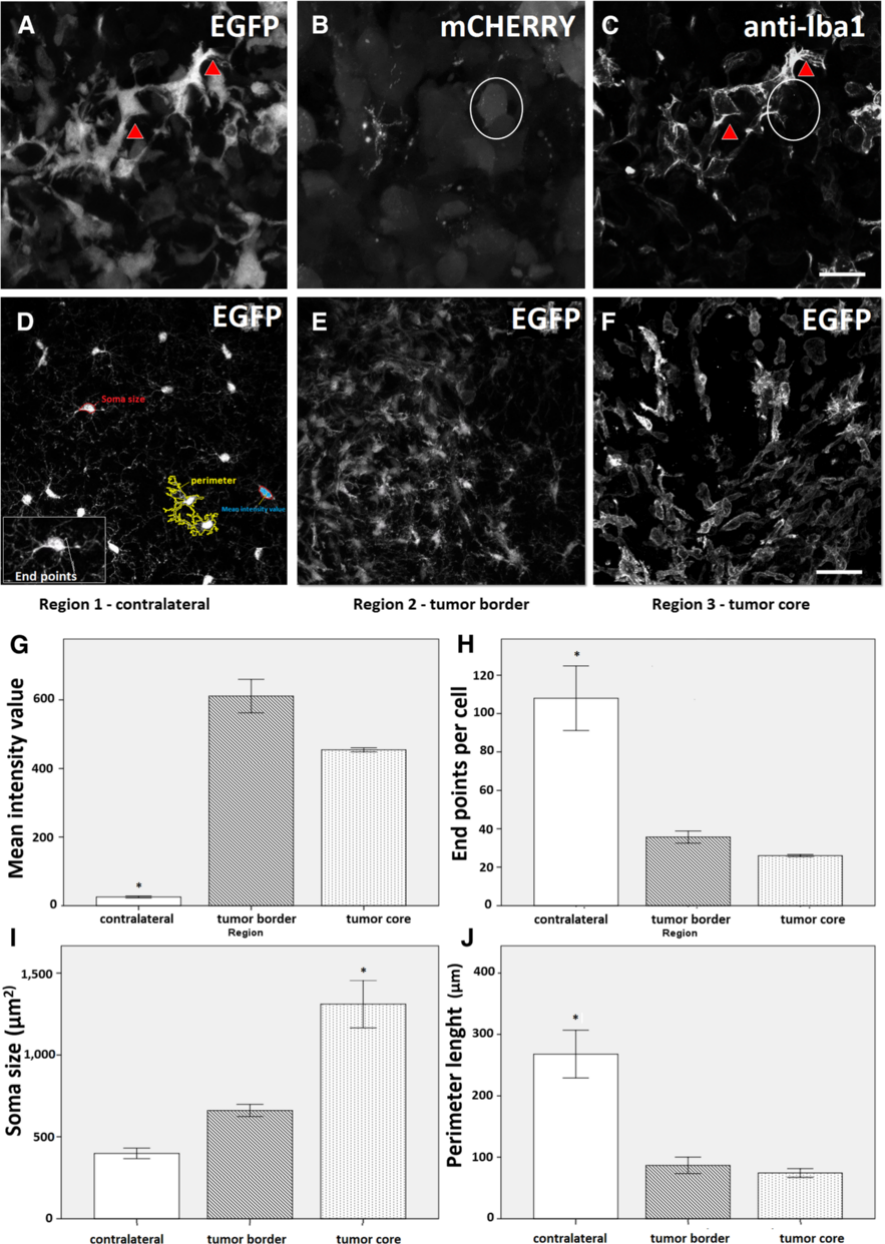
Quantitative morphometrical analysis of microglial activation during glioma growth reveals regional differences between core, border and control regions. Images from cortical regions implanted with glioma cells at 14 dpi. **a** Endogenous EGFP fluorescence in microglial cells (red arrowheads) in the tumor core in relation to (**b**) GL261 cells labeled with mCherry (*white circle*) were immunopositive for the microglial/macrophage marker Iba1 (**c**, *red arrowheads*). **d**–**f**) EGFP channel of representative slices showing morphological changes in microglia, from the non-inoculated region 1 (**d**) to the tumor regions 2 (**e**) and 3 (**f**). In (**d**), a set of parameters for morphometrical analysis of individual microglial cells is represented as follows. Blue filled in red outline: mean intensity value of Iba1 expression (**g**); End points, the ImageJ plugin processed Skeleton tags of all pixel/voxels in a skeleton image. All junctions were classified in different categories depending on their 26 neighbors. When they had less than 2 neighbors, they were counted as end-points voxels (**h**); Red contour: soma size measured in μm^2^ (I); yellow contour: total perimeter length in μm (**j**). Data were expressed as mean ± SEM. (*p < 0.05 statistical significance, ANOVA one way followed by Tukey’s test). Scale bar: 20 μm

We examined microglial cells at 14 dpi to quantify parameters of microglial activation. First, we confirmed that EGFP-positive cells of the GL261/CX3CR1 glioma model express Iba1 (Fig. 3a–c). Iba1 is a marker of microglia/macrophages and can also indicate the activated status of microglia. Iba1 expression was higher in tumor regions in comparison with the contralateral hemisphere. The mean intensity values of anti-Iba1 were 611.4 ± 49.0 and 453.0 ± 6.0 in tumor border (Fig. 3e) and tumor core (Fig. 3f), respectively, in comparison with 24.8 ± 2.8 found in non-inoculated sites (Fig. 3d, g; *p* < 0.05).

To quantify the changes in microglial morphology, we used maximum intensity projections of confocal images (EGFP channel). We first determined the number of endpoints per cell, that represents the branch ends. Microglial cells in the border or the tumor core showed less end points (35.7 ± 3.1 and 26.0 ± 0.5, respectively) in comparison with those in the contralateral control hemisphere (108.0 ± 16.8) (Fig. 3h; *p* < 0.05). Microglial cells in the tumor core region also showed an enlarged soma size (1310 ± 146.0 μm^2^) in comparison with those in tumor borders and the contralateral hemisphere (661 ± 37.4 μm^2^ and 366 ± 0.0 μm^2^, respectively) (Fig. 3i; *p* < 0.05). Finally, the cell perimeter lengths in tumor regions (74.3 ± 7.0 μm in tumor center and 86.7 ± 13.4 μm in the border) were smaller than those found in the contralateral control site (268.0 ± 38.8 μm) (Fig. 3j; *p* < 0.05).

In summary, our morphometric results showed that microglial cells apart from glioma tissues have a more ramified morphology. They present a higher number of thin processes extending away from their small soma. When growing next to tumor regions, cells reduced the number of processes and enlarged their soma, assuming a typical amoeboid shape. These data suggest that glioma tumors influence microglial cells to become activated and change their morphology.

### Tracking the time-course of microglia and glioma cell interactions

**Fig. 4:**
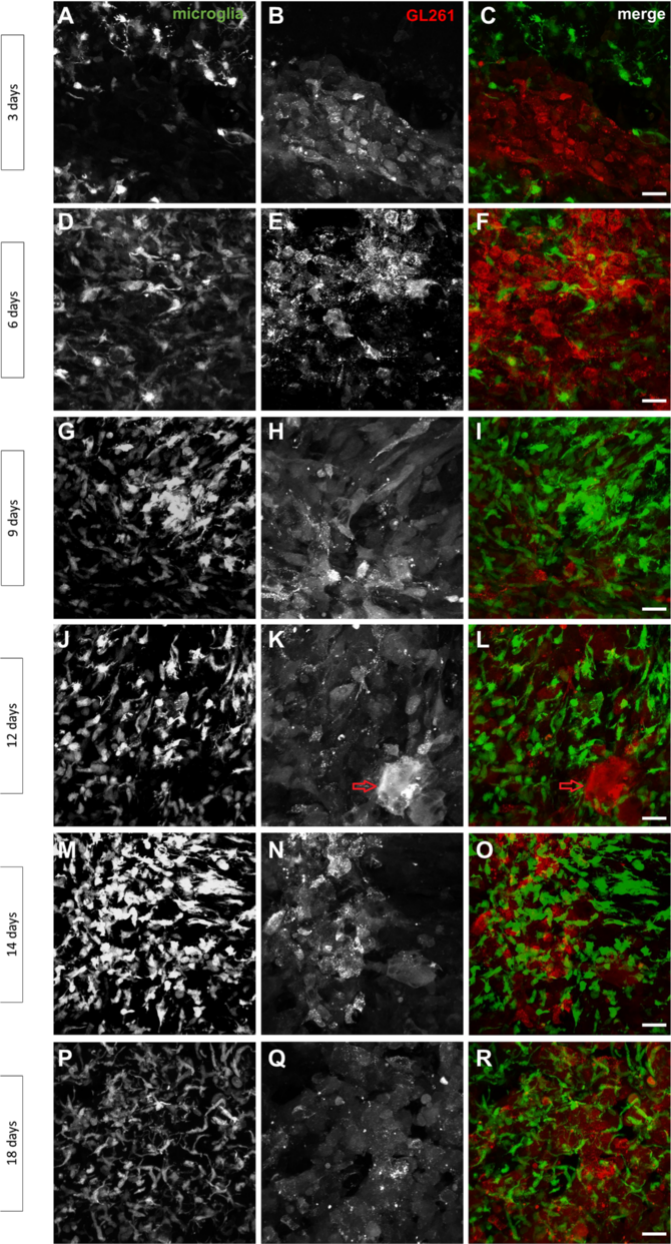
Observation of microglial and tumor cells reveals morphological changes in the core during gliomagenesis. **a**–**r** Time-course of glioma growth in mouse brain implanted with mCherry-GL261 cells 3, 6, 9, 12, 14 and 18 days after mCherry-GL261 injection in adult TgH(CX3CR1-EGFP) mice shown as maximum intensity projections of the tumor core. Channels are represented by endogenous fluorescence of EGFP in microglia (*left column*), mCherry in GL261 glioma cells (*middle column*) and merge (*right column*). As shown in K and L (*red arrow*), a polykaryocyte cell appeared at 12 dpi. Scale bar: 20 μm

The time-course of microglial growth in tumor regions was examined by confocal laser scanning microscopy at 3, 6, 9, 12, 14, and 18 dpi (Fig. 4a–r). At 3 dpi, we found activated microglia (in green) surrounding glioma mCherry-GL261 cells without infiltrating the tumor mass (Fig. 4a, b, c). From 6–18 dpi, green fluorescent microglial cells infiltrated the tumor mass (in red), as shown in merge images of Fig. 4f, i, l, o, r. Indeed, cells in close contact with glioma presented a typical activated morphology, with amoeboid shape and pseudopodia (Fig. 4, right column).

Diameter and mean intensity value of glioma cells were determined by using the ImageJ plugin ROI. For each time point, 10 cells (mCherry channel) per image, of three animals were randomly analyzed. mCherry-GL261 tumor cells displayed significant differences in cell morphology during their growth. At 3 dpi, they showed similar sizes and shapes, presenting a uniform and round shaped morphology (Fig. 4b); diameters of cell bodies ranged from 10–20 μm. Tumors increased in size in later stages, and glioma cells assumed clear morphological changes, as occurred at 14 dpi (Fig. 4n). The tumor core contained a population of cells heterogeneous in size and morphology; at this stage, diameters of glioma cell bodies varied from 14–52 μm. Cells also differed in fluorescence intensity as the tumor grows, ranging from 167 at 3 dpi to 626 at 14 dpi. In addition, we found an another cell type in tumor region at 12 dpi. They were polykaryocytes, i.e. multinucleated cells, with a bizarre nuclei structure and enlarged cytoplasm up to 60 μm of diameter (Fig. 4k, l).

### In vivo imaging by 2P-LSM reveals the kinetics of microglial interaction with glioma cells

**Fig. 5:**
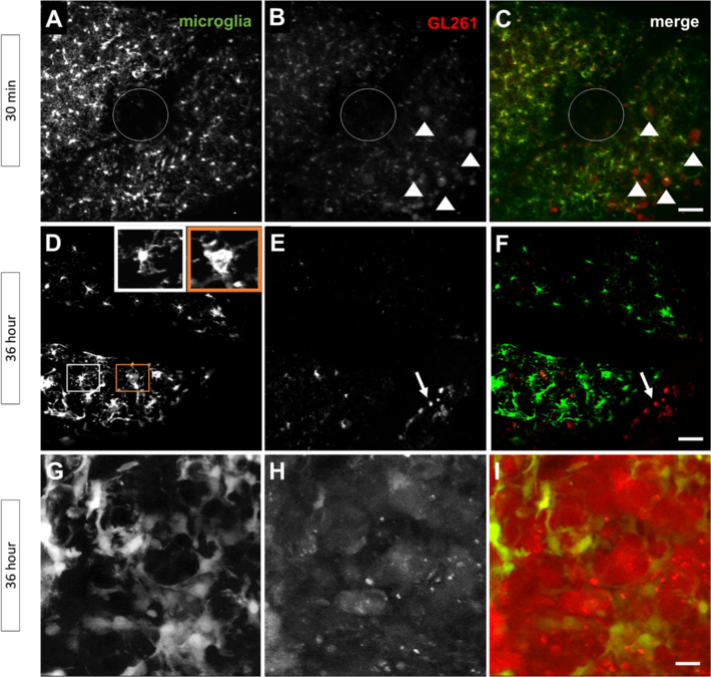
Two-photon laser-scanning microscopy (2P-LSM) reveals cellular responses of microglia to tumor injections in vivo in TgH(CX3CR1-EGFP) mice. **a**–**c** Microgliosis around the tumor injection site (*circle*), 30 min after implantation. White triangles point to mCherry-GL261 cells infiltrating the brain cortex. **d**–**f** At 36 h, microglial cells presented either a typical amoeboid shape (*orange squares*, **d**) or a ramified morphology, with a small cell body and long processes (*white squares*, **d**). mCherry-GL261 cells were present in capillary borders, suggesting a route for cancer cell spreading (*white arrows*, **e** and **f**). **g**–**i** Microglia remained infiltrated and in close contact with tumor cells, suggesting a cell-cell communication during tumor growth. Scale bars: (**a**–**f**), 50 μm; (**g**–**i**), 20 μm

The GL261/CX3CR1 model proposed in our study also provided brain tumor images of living animals. A 2P-LSM microscopy captured the fluorescence emitted by microglia and glioma cells during tumorigenesis, allowing an intravital- and noninvasive imaging. First, we could track the early stages of microglial activation. Already 30 min after injection, we noted microglia (Fig. 5a) in close contact with mCherry-GL261 cells (Fig. 5b and c, white triangles showing glioma cells). At 36 h, microglial cells presented different morphologies, as shown in Fig. 5d, squares. Some cells were typically amoeboid (Fig. 5d, orange outlined square); others had a ramified morphology, with small processes (Fig. 5d, white outlined square).

Regarding mCherry-GL261 cells, they infiltrated into the brain parenchyma and, in addition, were present at the margins of blood capillaries (Fig. 5e and f, white arrows). This anatomic location favors the metastatic behavior of glioma cells cited above (see Fig. 1c). At 36 h after injection, microglia remained growing in close contact with tumor cells (Fig. 5g, h and i).

## Discussion

Glioblastoma multiforme (GBM) is the most aggressive and difficult to treat brain tumor [24, 25]. To better understand glioma biology, studies have taken advantage of various animal models [14, 15, 26]. The cell line GL261 is a well-established model of GBM, developed in 1939 by chemical induction with methylcholanthrene in C3H mice [27]. Injection of GL261 tumor fragments in a syngeneic host caused a glioma. A permanent GL261 cell line was obtained in the 1990s [28]. Since then, GL261 cells have been used in research about immunotherapy and, in addition, in many studies addressing tumor biology [29]. The orthotopic GL261 mouse model displays key features also found in human GBM. Cells are similar in morphology, invasive behavior and histopathological markers, presenting mutations and deregulated signaling pathways [7, 8]. In our study, mCherry-GL261 cells injected into brain areas showed the typical aggressive and metastatic behavior found in patients with GBM. Three days after injection, we could note a mass of cells developing rapidly (Fig. 1). In the following days, tumor mass increased in size, compressing the hippocampus. Indeed, it spread cells to form secondary tumors in brain areas distant from the injection site, like the hypothalamus (Fig. 1c, arrow). mCherry-GL261 cells were also found next to capillaries, suggesting blood vessels were used to spread tumor cells (Fig. 5e, f).

Besides recapitulating most features of human GBM, the GL261 glioma model also presents a remarkable experimental advantage − tumor grows in immunocompetent animals [8]. Considering the biological question addressed in our study, this feature was strikingly relevant. We could examine the role of cells of the innate immune system, the microglia, in gliomas growing without immunosuppression. GL261 cell line used in our work was developed to express the mCherry fluorescence [19]. In a previous work, mCherry fluorescence was also applied to mark glioma cells. As the authors chose the U251 line, they had to use an immunossupressed mouse line [30].

Imaging of microglial cells was a critical issue in our study. In our model of glioma, we chose the TgH(CX3CR1-EGFP) mouse line, based on immunocompetent animals engineered to show fluorescent microglial cells. This mouse line was previously used to address the role of microglia in spinal cord development, during neurodegeneration and the early aspects of inflammatory response in the central nervous system [31–33]. Regarding gliomas, the TgH(CX3CR1-EGFP) mouse strain was used to investigate aspects of tumor biology, like the role of CX3CR1 receptors in malignant glioma and a preclinical rationale for the development of stroma-directed glioma therapies in children [34–36].

Rio-Ortega was the first to recognize microglia as a distinct population of cells in the central nervous system. He found that microglia respond to brain injury by migrating to sites of tissue damage, where cells presented marked changes in their morphology [37]. In our GL261/CX3CR1 model, microglial cells expressing EGFP allowed us to analyze and quantify that morphological changes described by Rio-Hortega, but now during glioma development . In addition, the model allowed us to track microglial migration toward the tumor mass, and to examine the respective changes in cell morphology. The surveilling microglia − with a ramified shape and found in non-inoculated areas − can become activated in tumor regions, adopting amoeboid shape.

Activation of microglia is an important change for restoring tissue integrity after injury of healthy tissues. This activation response will rapidly mitigate local infection or cell damage. During glioma development, however, microglia present a distinct role. Instead of starting the expected anti-tumor response, microglial cells switch to a pro-tumorigenic alternative phenotype. In such conditions, microglia contributes to tumor growth, invasion, and angiogenesis. Activated microglia also cause immunosuppression by releasing cytokines/chemokines and extracellular matrix proteases [38, 39]. This “Janus face” of microglia during gliomagenesis is still poorly understood. That results, in part, to the lack of methods able to quantify microglia activation longitudinally, in growing glioma tissues. Our study confirmed that expression of Iba1, enhanced in activated microglia, increases in tumor areas. It is a simple method to survey the cell activation in fixed brain tissues. In addition, our model also presents some advantages. It enables to examine the dynamics of microglial activation, related to changes in morphology and pattern of infiltration, after their recruitment by glioma. mCherry-GL261 cells induced changes in microglial morphology to an activated amoeboid shape. Amoeboid microglia interacted with glioma cells since the early stages of tumor growing, when glioma cells have shown a homogeneous shape. This close contact remained until the last time point of our study, 18 dpi, in which glioma tumor cells presented a highly diverse and diffuse morphology. A previous work has also reported the same change in microglia morphology in contact with glioma tumor, in brain sections obtained 15 dpi [40]. Indeed, results (as shown in Fig. 2b) corroborated the author’s finding that tumor areas presented a higher microglial density compared to control brain hemispheres.

Previous studies with the same GL261 mouse model have examined how microglia interact with glioma cells to affect tumor growing. Glioma cells could influence microglia to express a membrane type 1 metalloprotease, which contributed to tumor expansion [3]. The authors organized serial images of tumor brain areas to achieve a 3-dimensionally picture of microglial cells growing around an inside tumor mass. A further study explored the effects of ganciclovir, a drug that reduced microglia cell number in tumor tissues. The treatment caused a marked decrease in tumor growing, confirming that microglia contributes to gliomagenesis [41]. In common, both studies used a mouse model based on GFP-expressing GL261 cells. In addition, they detected microglial growing and tumor infiltration by Iba1 immunofluorescence. In our study, we also identified a pattern of microglia migration toward the tumor mass. First, activated microglia growth around the tumor mass, then the cells infiltrated the tumor tissue. Taking together, these data revealed that microglia and glioma cells grow in a well-organized and close spatial relationship during gliomagenesis.

Two-photon laser scanning microscopy (2P-LSM) has brought several advantages for intravital imaging of fluorescent cells [16]. Compared to confocal microscopy, the method offers a 100-fold increase in penetration depth, which is valuable for studies addressing brain tissues [17, 18]. Also, 2P-LSM discriminates fluorescent signals at a submicrometer scale, with enough signal intensity at increased depth in brain tissues [42]. As the method offers a true three-dimensional imaging in a living organism, sequential events can be recorded in the same specimen at extended periods. In neuroscience, 2P-LSM has allowed studies on nervous system development, cell physiology, plasticity, and neuron degeneration [43–48]. Intravital microscopy of rodents engineered to express cell-specific fluorescence has provided valuable insights into mammalian biology and the mechanism of diseases [49–51]. In this study, the non-invasive 2P-LSM enabled an in vivo imaging of activated microglia in brain tumors of living animals . We found activated microglia contacting glioma cells shortly after tumor seeding. In 30 min, activated microglia have established contact with mCherry-GL261 glioma cells.

## Conclusions

The present work evaluated a GL261/CX3CR1 model, dedicated to study microglial interaction with glioma tumors. The preparation allowed to track many features of microglial activation by glioma cells, and revealed new insights into cell-cell communication in an immunocompetent mouse. Microscopy techniques and new approaches for imaging analysis provided a quantitative assessment of microglial activation and migration toward the tumor mass. GL261/CX3CR1 revealed the close contact between microglia and glioma cells, from imaging techniques that present many advantages. Cell-cell interaction was monitored: 1- longitudinally; 2- in locus; 3- with cellular resolution; 4- from fixed tissues or living animals; 5- without the need of immunodetection; 6- on timescales from minutes to weeks. In our evaluation, the method provides a rapid unbiased technique, enabling analysis of large datasets of imaging volumes taken at multiple time-points. In conclusion, GL261/CX3CR1 is a valuable model for studies about glioma-microglia interactions.

## Abbreviations

2P-LSM: Two-photon laser scanning microscopy
CA1, CA2, CA3: *Cornu Ammonis* areas
CX3CR1: Fractalkine receptor
DMEM: Dulbecco’s modified eagle’s medium
dpi: Days post-injection
EGFP: Enhanced green fluorescent protein
GBM: Glioblastoma multiforme
GFP: Green fluorescent protein
Iba: Ionized calcium binding adaptor molecule 1
IgG: Immunoglobulin G
MT1-MMP: Membrane type-1 matrix metalloproteinase
NK: Natural killer cells
PBS: Phosphate-buffered saline
RT: Room temperature
